# Class IA Phosphatidylinositol 3-Kinase p110α Regulates Phagosome Maturation

**DOI:** 10.1371/journal.pone.0043668

**Published:** 2012-08-22

**Authors:** Emily P. Thi, Ulrike Lambertz, Neil E. Reiner

**Affiliations:** 1 Departments of Medicine, Experimental Medicine Program, Division of Infectious Diseases, University of British Columbia and the Immunity and Infection Research Centre, Vancouver Coastal Health Research Institute, Vancouver, B.C., Canada; 2 Microbiology and Immunology, University of British Columbia and the Immunity and Infection Research Centre, Vancouver Coastal Health Research Institute, Vancouver, B.C., Canada; Institut de Pharmacologie et de Biologie Structurale, France

## Abstract

Of the various phosphatidylinositol 3- kinases (PI3Ks), only the class III enzyme Vps34 has been shown to regulate phagosome maturation. During studies of phagosome maturation in THP-1 cells deficient in class IA PI3K p110α, we discovered that this PI3K isoform is required for vacuole maturation to progress beyond acquisition of Rab7 leading to delivery of lysosomal markers. Bead phagosomes from THP-1 cells acquired p110α and contained PI3P and PI(3,4,5)P3; however, p110α and PI(3,4,5)P3 levels in phagosomes from p110α knockdown cells were decreased. Phagosomes from p110α knock down cells showed normal acquisition of both Rab5 and EEA-1, but were markedly deficient in the lysosomal markers LAMP-1 and LAMP-2, and the lysosomal hydrolase, β-galactosidase. Phagosomes from p110α deficient cells also displayed impaired fusion with Texas Red dextran-loaded lysosomes. Despite lacking lysosomal components, phagosomes from p110α deficient cells recruited normal levels of Rab7, Rab-interacting lysosomal protein (RILP) and homotypic vacuole fusion and protein sorting (HOPs) components Vps41 and Vps16. The latter observations demonstrated that phagosomal Rab7 was active and capable of recruiting effectors involved in membrane fusion. Nevertheless, active Rab7 was not sufficient to bring about the delivery of lysosomal proteins to the maturing vacuole, which is shown for the first time to be dependent on a class I PI3K.

## Introduction

Phagosome maturation is a progressive process, in which a nascent phagosome sequentially interacts with early and late endosomes, resulting in the formation of structures termed early and late phagosomes, respectively. The eventual interaction of late phagosomes with lysosomes results in the formation of phagolysosomes and ideally, the degradation of phagosomal contents by lysosomal hydrolytic enzymes. Regulation of these events is thought to mainly involve two groups of enzymes, namely phosphatidylinositol 3- kinases (PI3Ks), and the small Rab GTPases. PI3Ks are lipid kinases that catalyze the phosphorylation of the 3′-hydroxyl group of phosphatidylinositol and phosphatidylinositides (PIs). PIs regulate many cellular signaling pathways affecting functions as diverse as cellular metabolism, cytoskeletal dynamics, and vesicle trafficking amongst others. One PI in particular, phosphatidylinositol 3-phosphate (PI3P), has been shown to play an important role in regulating the recruitment of various effector proteins essential to phagosome maturation. Although all three classes of PI3Ks can either directly or indirectly produce PI3P, only the class III enzyme Vps34 has thus far been implicated in phagosome maturation.

The maturation process is thought to be influenced by the ligands and receptors engaged in phagocytosis, although only very recently have there been detailed studies examining the effects of specific ligand-receptor interactions on phagosome maturation [1], [2]. Phagocytic receptors may be classified into three groups: opsonic receptors, such as the F_c_γR and complement receptors, non-opsonic receptors (mannose receptor, and other scavenger receptors), and receptors which may serve as co-receptors or co-stimulatory molecules in the recognition of pathogen associated molecular patterns (such as TLRs, and the recently characterized microbial sensor SLAM [3]–[5].

The contribution of opsonic receptors such as F_c_γR-mediated uptake of IgG-opsonized prey to phagosome maturation has been well documented [6]–[8]. In contrast, the role of TLRs in influencing phagosome maturation is controversial [4], [5], while the recently characterized SLAM protein appears to play a role in regulating NOX2 activity and may serve to link the maturation of phagosomes containing Gram negative prey with the autophagy pathway [3]. The effects on phagosome maturation in response to complement receptor mediated phagocytosis have not been studied as thoroughly. It is known that *Mycobacterium tuberculosis* is taken up primarily through CR3 [9], [10]. It has been postulated that maturation arrest of the *M. tuberculosis* vacuole is necessary for its intracellular survival and that this may be dependent on modulation of Ca^2+^ signalling events that are triggered by CR3 uptake [11]–[13].

Taken together, these studies [1]–[5], [7], [8], [11]–[15] suggest that the precise pathways involved in phagosome maturation likely vary depending on the receptors engaged by the phagocytic prey. In light of this, we were interested in assessing whether the dogma of Vps34 being the only PI3K involved in phagosome maturation was also valid in the case of prey taken up by receptors other than the FcγR. To this end, we examined phagosome maturation in a human monocytic cell line in which expression of the catalytic subunit of a class IA PI3K, p110α, was selectively silenced by two independent lentiviral systems [16]. These cells were either infected with a representative biological prey (non-pathogenic bacterium *Mycobacterium smegmatis*, which is taken up mainly by CR3 and mannan receptors [10], [14], [17]–[19]) or fed BSA-coated magnetic beads as non-specific prey. Our results identify a novel and previously unknown function for this class IA PI3K in regulating late events in phagosome maturation. p110α appears to function in parallel to Rab7 activation and translocation to the phagosome membrane. We show that the presence of Rab7 is insufficient to mediate the acquisition of lysosomal components in the absence of the PI3K p110α. Our findings advance the novel concept that other classes of PI3K in addition to Vps34 regulate phagosome maturation.

## Results

### Knockdown of the Class IA PI3K p110α does not Impair Phagocytosis of Prey Less than or Equal to 3 µm in Diameter

**Figure 1:**
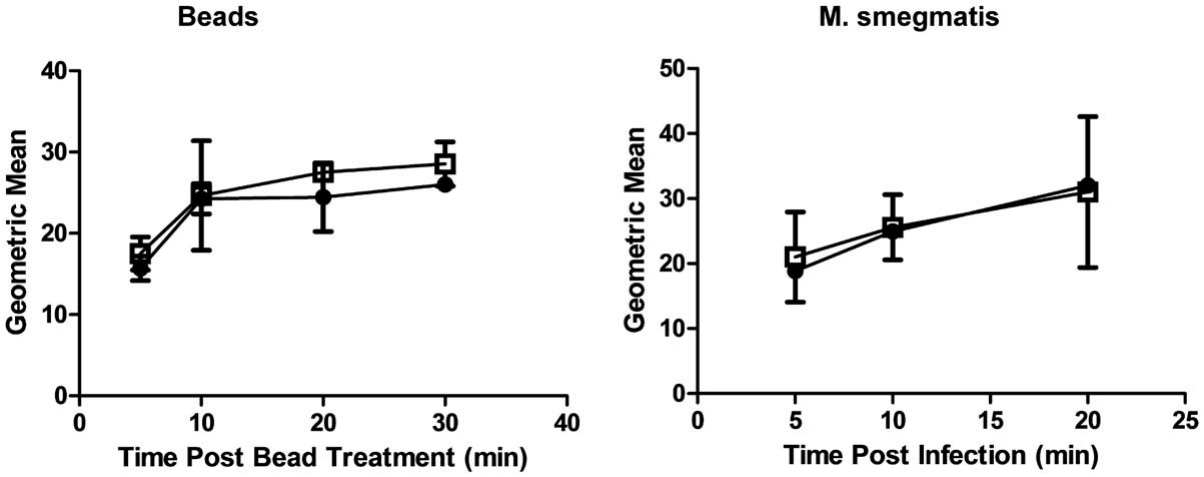
Knockdown of PI3K p110α does not impair phagocytosis of prey less than 3 µm in diameter. Cells were either fed Alexafluor 633-SE labelled BSA-coupled 3 µm magnetic beads or infected with Alexafluor 633-SE labelled *M. smegmatis.* Flow cytometric analysis was done to determine uptake. Solid circles  =  control cells, empty squares  =  p110α knockdown cells. Data are means +/− s.e.m., from three independent experiments. No significant differences were observed.

Selective, stable knockdown of expression of the class IA PI3K p110α in the THP-1 human monocytic cell line was done by lentiviral transduction as described previously [16]. It has been documented that inhibition of PI3K activity impairs phagocytic uptake of prey larger than 4 µm [20]. To test the ability of constitutive p110α knockdown cells to take up prey less than or equal to 3 µm in diameter, we fed phorbol-12-myristate-13-acetate (PMA)-differentiated control and p110α knockdown cells BSA-coupled 3 µm magnetic beads. Alternatively, parallel cell cultures were infected with the minimally virulent, opportunistic organism *M. smegmatis*. Prey, both beads and bacteria, were covalently surface labelled with the fluorescent dye Alexafluor 633 succinimidyl ester (-SE) to allow quantitation of uptake by flow cytometry. Both control and p110α constitutively deficient cells displayed equivalent levels of uptake (Fig. 1), thereby indicating that knockdown of p110α did not impair phagocytosis of the prey involved in this study.

### Phagosomes from p110α Knockdown Cells have Less p110α and PI(3,4,5)P3 but Similar Levels of PI3P

**Figure 2:**
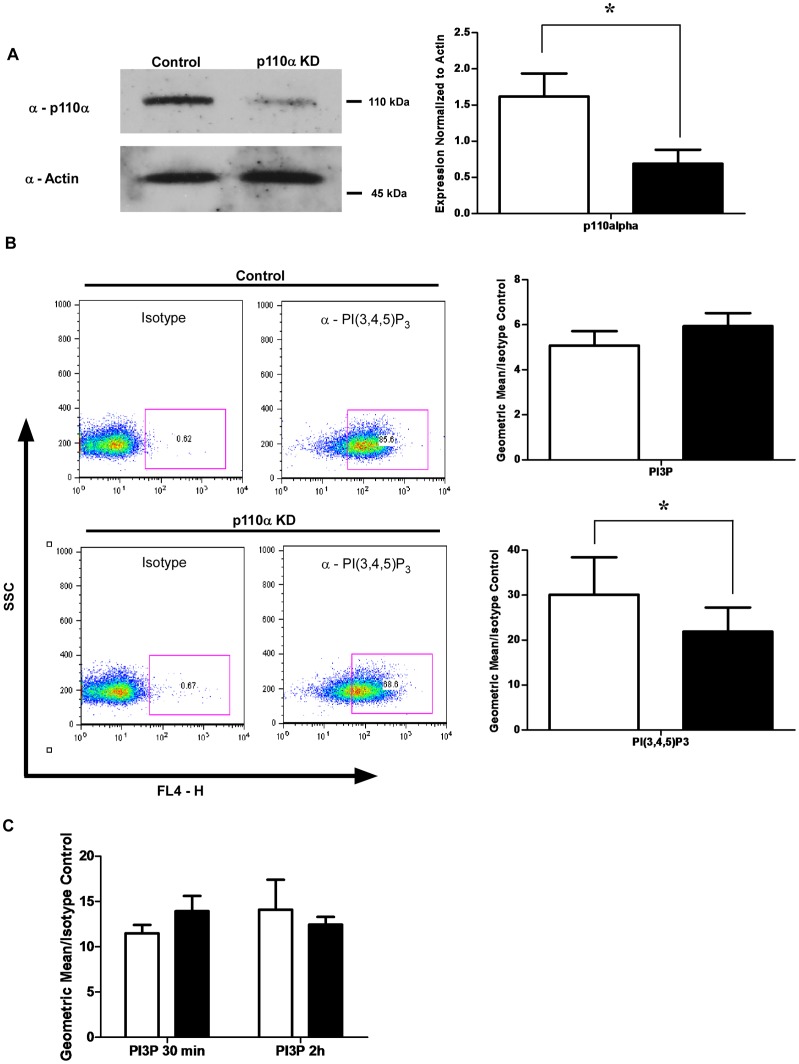
Phagosomes contain the class IA PI3K p110α, and display PI(3,4,5)P_3_. Phagosomes from p110αknockdown cells show decreased p110α recruitment and PI(3,4,5)_3_ levels. **A)** *Phagosomes isolated from PMA-differentiated THP-1 cells contain the class IA PI3K p110α, and this is decreased in knockdown cells.* Late stage 4 h bead phagosomes were isolated by magnetic pulldown, lysed, and immunoblotted for p110α. Actin was done as a loading control. Densitometry of four independent experiments indicates a significant decrease in phagosomal p110α in phagosomes isolated from knockdown cells. Empty bars  =  control cell phagosomes, solid bars  =  phagosomes from p110α knockdown cells. p<0.05 **B)** *Phagosomes from p110α knockdown cells contain less PI(3,4,5)P3, but similar amounts of PI3P as control cells.* Bead phagosomes were isolated by magnetic pulldown, fixed, stained for PI3P and PI(3,4,5)P3, and flow organellometry was done. A representative dot plot of SSC vs FL4 fluorescence channel depicting how the phagosomes were gated is shown. The data shows PI(3,4,5)P3 staining of 4 h phagosomes isolated from control and p110α knockdown cells from one experiment. Empty bars  =  control cell phagosomes, solid bars  =  phagosomes from p110α knockdown cells Data are means +/− s.e.m., from four independent experiments. The levels of PI(3,4,5)P3 were found to be significantly decreased in phagosomes isolated from p110α deficient cells. p<0.05. No significant differences were observed for PI3P levels. **C)** *Kinetics of PI3P acquisition onto phagosomes.* Phagosomes were isolated at 30 min and 2 h and stained for PI3P. The graph shows geometric means normalized to isotype control. Open bars  =  phagosomes isolated from control cells, Solid bars  =  phagosomes isolated from p110α knockdown cells. No significant differences were observed, and PI3P levels appeared to remain consistent throughout phagosome maturation.

We examined whether p110α is recruited to phagosomes and assessed phagosomal levels of its phosphoinositide product, PI(3,4,5)P3. PMA-differentiated control and constitutive knockdown cells were fed BSA-coupled 3 µm magnetic beads and 4 hour phagosomes were isolated by magnetic pulldown. Phagosomes were then either fixed for staining with antibodies against PI3P and PI(3,4,5)P3, or lysed for determination of p110α phagosomal recruitment by immunoblotting. Our results showed that the PI3K p110α was associated with phagosomes isolated from THP-1 cells, and that the class IA PI3K phophoinositide product, PI(3,4,5)P3 was also present on these phagosomes. Phagosomes isolated from p110α knockdown cells displayed significantly less recruitment of this PI3K (Fig. 2A), and a decreased amount of PI(3,4,5)P3, though levels of PI3P were unaffected (Fig. 2B). Flow organellometry of bead phagosomes indicated that PI3P levels appeared to be unchanged throughout phagosome maturation (Fig. 2C).

### Flow Organellometry of Phagosomes from p110α Knockdown Cells Shows Similiar Levels of Rab5B and EEA-1, but a Defect in Acquisition of Lysosomal Marker Proteins

**Figure 3:**
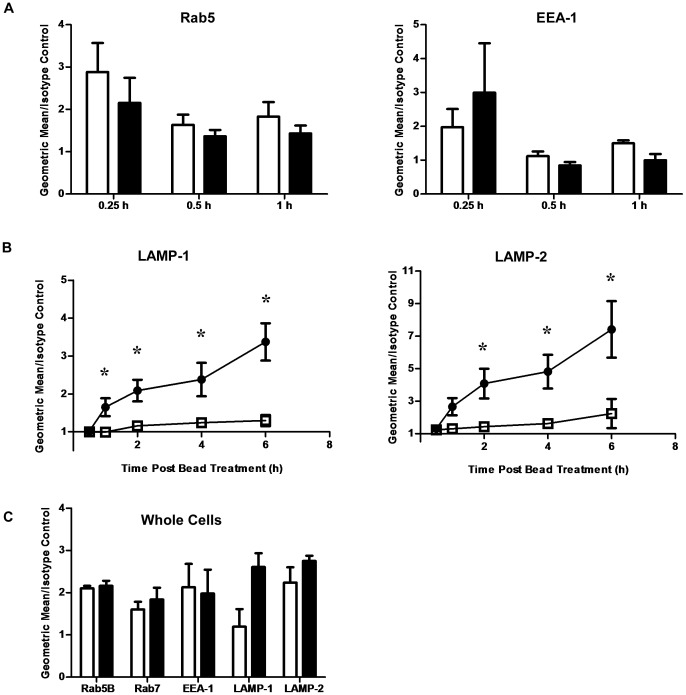
Magnetic bead phagosomes from p110α knockdown cells show defective acquisition of the lysosomal proteins LAMP-1 and LAMP-2. Kinetic analysis of phagosome maturation by flow organellometry shows **A)** normal acquisition of the early endosomal marker Rab5 and EEA-1, and **B)** marked defects in the acquisition of the lysosomal membrane proteins LAMP-1 and LAMP-2 by p110α knockdown cells. Solid circles  =  control cells, empty squares  =  p110α knockdown cells. Timepoints were taken at 30 minutes, 1 hour, 2 hours, 4 hours, and 6 hours post bead treatment. Data are means +/− s.e.m., from five independent experiments. Ratios of geometric mean over isotype control of 1 or less are interpreted as negligible signal. *p<0.05. **C)** Whole cell expression of phagosome markers are at similar levels in control and p110α knockdown cells. Staining of permeabilized cells and flow cytometric analysis of various phagosome markers in control and p110α knockdown cells showed no significant differences in cellular levels. Empty bars  =  control cells, solid bars  =  p110α knockdown cells. Data are means +/− s.e.m., from three independent experiments.

Control and constitutive p110α knockdown cells were fed BSA-coupled 3 µm magnetic beads and phagosomes were isolated at various timepoints. Vacuoles were then stained for various phagosome maturation markers and analyzed by flow organellometry (Fig. 3). p110α knockdown cells acquired similar levels of the early endosomal small GTPase, Rab5B, and early endosomal autoantigen 1 (EEA-1) (Fig. 3A). However, acquisition of LAMP-1 and LAMP-2 by p110α deficient cells was markedly impaired (Fig. 3B). Nevertheless, both cell types were found to express similar levels of these markers at the whole cell level (indeed, the knockdown cells appear to express increased cellular levels of LAMP-1) (Fig. 3C). Furthermore, confocal fluorescence microscopy showed no defects in biosynthetic sorting of LAMP-1 from the trans-Golgi network to the endosomal system (data not shown). Thus, deficient acquisition of these lysosomal membrane proteins may have been the result of null fusion events between maturing phagosomes, endosomes and lysosomes. Our kinetic studies which consisted of a relatively long time course (up to 6 hours) also indicate that this defect in acquiring LAMP proteins was not simply due to delayed kinetics, but an actual block in marker acquisition.

### Confocal Analysis of p110α Knockdown Cells Showed Impaired Phagosomal Acquisition of LAMP-1 and a Defect in Phagosome Fusion with Dextran-loaded Lysosomes

**Figure 4:**
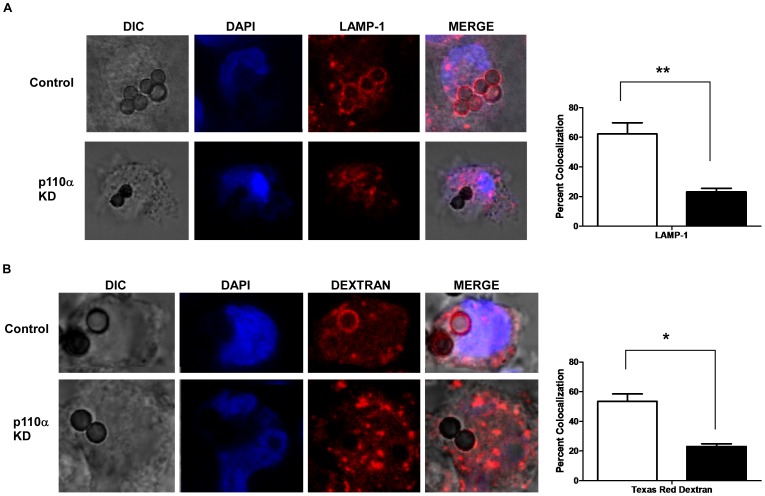
Phagosomes from p110α knockdown cells show marked decreases in LAMP-1 acquisition and phagolysosome fusion. **A)** Confocal microscopy of control and p110α knockdown cells shows reduced phagosomal LAMP-1 acquisition. The graph to the right shows counts of phagosomes colocalized with LAMP-1 staining. At least 100 phagosomes in each of three independent experiments were counted. Open bar  =  Control cells, Solid bar  =  p110α knockdown cells. Means +/− s.e.m. are shown. **p<0.01. **B)** Confocal microscopy of control and p110α knockdown cells shows reduced phagosome - lysosome fusion. Cells were loaded with Texas Red dextran, which was then chased into lysosomes. The graph to the right shows the numbers of phagosomes that were positive for a dextran signal. At least 100 phagosomes in each of three independent experiments were counted. Open bar  =  control cells, Solid bar  =  p110α knockdown cells. Means +/− s.e.m. are shown. *p<0.05.

In order to confirm a defect in LAMP-1 acquisition, cells were fed beads and assessed for LAMP-1 recruitment to bead-containing phagosomes. Confocal microscopy showed that p110α deficient cells had a significant reduction in LAMP-1 acquisition, in close agreement to our flow organellometry results (Fig. 4A). In addition, phagosomes from p110α knockdown cells show a significant decrease in fusion with lysosomes which had been loaded with Texas Red dextran, thus indicating abrogation of phagolysosome fusion (Fig. 4B). This result correlated well with our LAMP-1 confocal microscopy data, and strongly suggests that phagosomes from p110α deficient cells have markedly reduced access to lysosomal components.

### Phagosomes from p110α Knockdown Cells Display Decreased Acquisition of the Lysosomal Hydrolase, β-galactosidase

**Figure 5:**
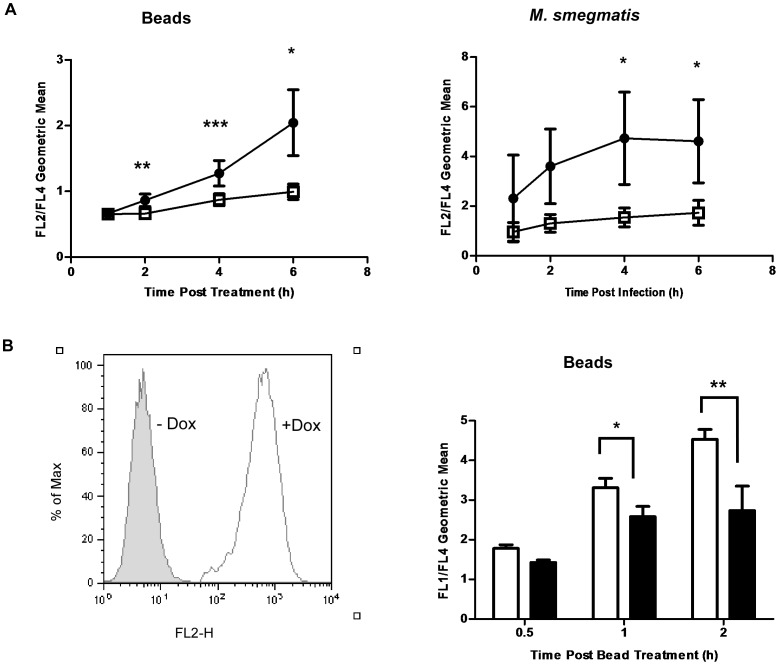
Delivery of the lysosomal enzyme β- galactosidase is impaired in p110α knockdown cells. **A)** Knockdown of p110α results in decreased acquisition of the lysosomal enzyme β- galactosidase by phagosomes containing either BSA coated beads or M. smegmatis. Cells were fed prey that had been previously surface labelled with Alexafluor 633-SE and the fluorescent β-galactosidase substrate, C_12_RG. Cleavage of C_12_RG releases fluorescence which is read in the FL2 channel of the flow cytometer. This was then normalized to the Alexafluor 633 signal (FL4) for phagocytic uptake. Solid circles  =  control cells, empty squares  =  p110α knockdown cells. Timepoints were taken at 1 hour, 2 hours, 4 hours, and 6 hours post bead treatment or infection. *p<0.05, **p<0.001, **p<0.0005. Data are means +/− s.e.m., from at least four independent experiments. **B)** Inducible p110α knockdown via a distinct shRNA sequence also showed defects in β-galactosidase acquisition. Knockdown was induced by doxycycline treatment prior to experimentation, which also induces expression of TurboRFP which is linked to shRNA production. The histogram on the left shows induction of red fluorescence when cells were given doxycycline. Cells were also fed the green fluorescent β-galactosidase substrate, C_12_FDG, which is read in the FL1 channel. Empty bars  =  Control cells (no doxycycline induction), Solid bars  =  Cells induced for p110α knockdown. *p<0.05, **p<0.001. Data are means +/− s.e.m. from five independent experiments.

In light of the deficient procurement of lysosomal membrane marker proteins on phagosomes isolated from p110α knockdown cells, it was of interest to know whether this was indicative of a broader defect extending to the delivery of active lysosomal enzymes. To examine this question, control and constitutive knockdown cells were assessed for the phagosomal acquisition and activity of the lysosomal hydrolase β-galactosidase in an assay developed by Yates et al. [21]. PMA-differentiated knockdown and control cells were fed either BSA-coupled 3 µm magnetic beads or infected with *M. smegmatis*. In both cases, prey had been previously labelled with Alexafluor 633-SE and a fluorescent β-galactosidase substrate, C12RG or C12FDG. Cleavage of the C12RG/C12FDG substrate on the surface of internalized prey by β-galactosidase and subsequent release of fluorescence occurs only when active lysosomal hydrolase is delivered to phagosomes containing labelled prey. Flow cytometric analyses of β-galactosidase activity of control and constitutive p110α knockdown cells showed that p110α knockdown cells displayed a marked reduction in the acquisition of β-galactosidase to phagosomes, and this was true for both beads and *M. smegmatis* prey (Fig. 5A). This defect in β-galactosidase delivery was also seen in cells in which p110α expression had been knocked down by a different, inducible shRNA sequence (Fig. 5B). The reduced delivery and activity of lysosomal β-galactosidase to phagosomes in p110α knockdown cells correlated well with our lysosomal marker data, and provided further evidence that p110α-deficient cells display a block in phagosome-lysosome fusion.

### Cathepsin D Delivery and Processing is Unaffected in Phagosomes Isolated from p110α Deficient Cells

**Figure 6:**
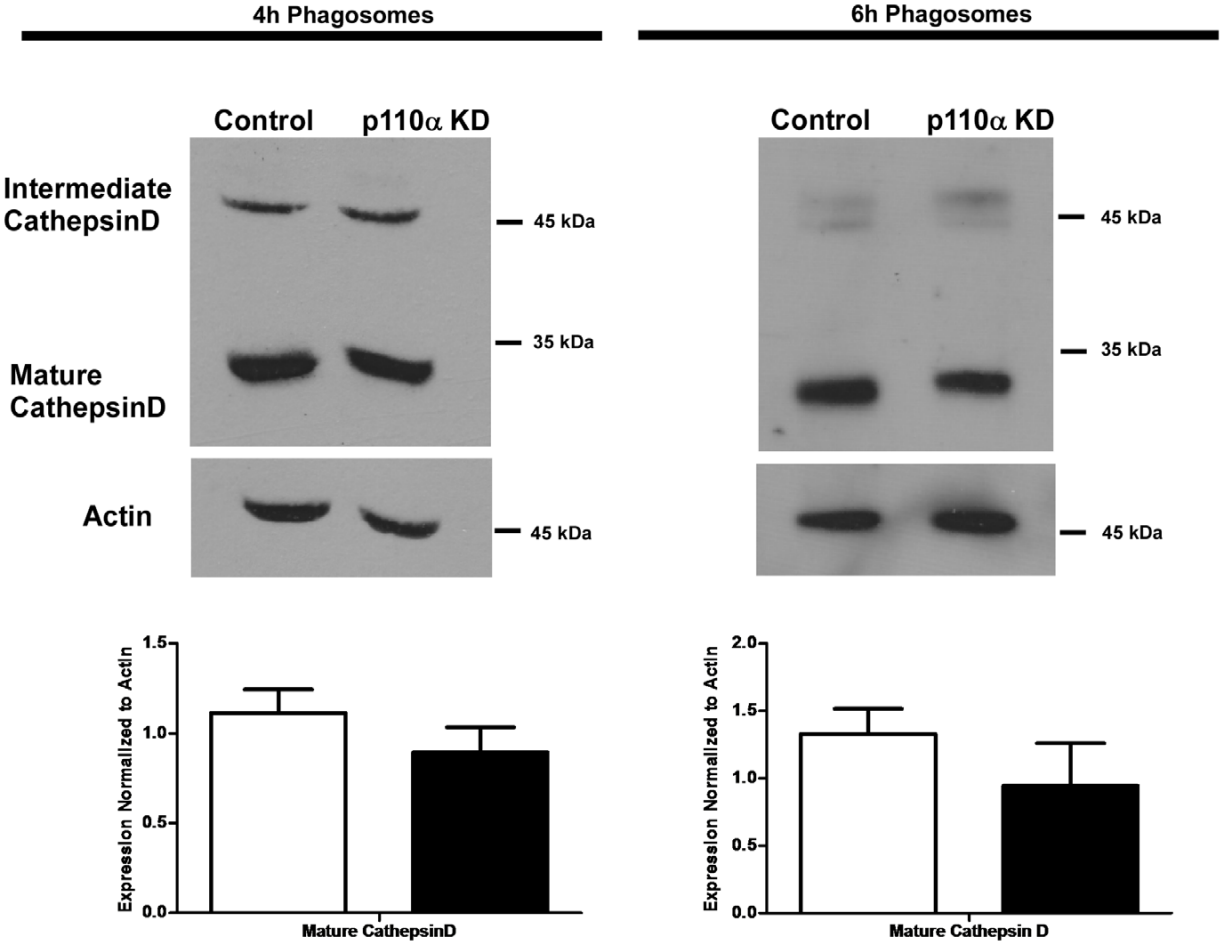
Phagosomes isolated from p110α knockdown cells show normal recruitment and processing of the protease cathepsin D. Lysates from 4 and 6 hour phagosomes were prepared, separated by SDS-PAGE and transferred to nitrocellulose membrane. Blots were probed with an antibody for cathepsin D. Actin is shown as a loading control. Although a trend towards less cathepsin D in the phagosome lysates from p110α knockdown cells was observed, it was not found to be statistically significant. Empty bars  =  control cells, solid bars  =  p110α knockdown cells. Data are means +/− s.e.m., from four independent experiments.

Delivery of cathepsin D is used as a marker of phagosome maturation [22]–[25]. To examine cathepsin D delivery to and processing in phagosomes isolated from p110α deficient cells, we probed 4 and 6 hour phagosome lysates by immunoblotting for cathepsin D using an antibody that recognizes all the processed forms. Compared with control cells, the results showed similar levels of both the intermediate and mature forms of cathepsin D, although there was a statistically non-significant trend towards lower levels of cathepsin D in the p110α knockdown cells. These results indicate that this protease is normally conveyed to and processed by phagosomes from p110α deficient cells (Fig. 6). The fact that cathepsin D was processed normally in p110α deficient cells suggested that phagosomal acidification was taking place, as acidification is needed to initiate proper processing of this pre-proenzyme [26]. Indeed, assessment of the acidification state of phagosomes from control and p110α deficient cells by infecting cells with *M. smegmatis* that had been previously surface labelled with the aciditropic dye, pHrodo™, showed no significant differences in acidification kinetics (data not shown), despite the concomitant lack of LAMP and hydrolase acquisition.

### Phagosomes from p110α Knockdown Cells Acquire Active Rab7 that is Competent in Mediating Recruitment of RILP and HOPS Components

**Figure 7:**
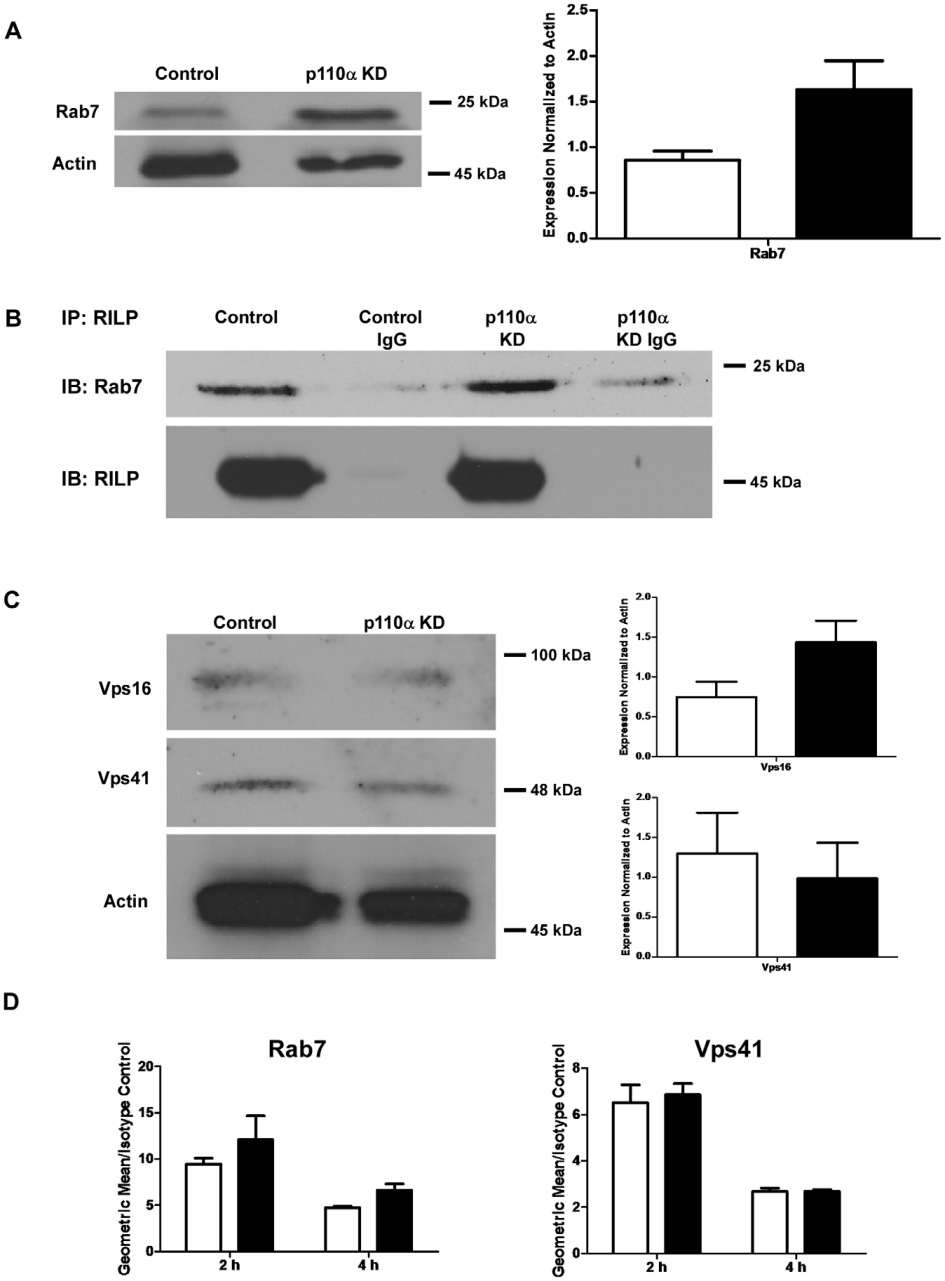
Phagosomes from p110α deficient cells recruit Rab7 and its effectors, RILP and HOPS. **A)** *Phagosomes from p110α knockdown cells are not defective in the recruitment of Rab7*. Western blots of bead phagosome lysates (4 hours) isolated from control and p110α knockdown cells show no significant differences in recruitment of Rab7. Actin is shown as a loading control. Empty bars  =  control cells, solid bars  =  p110α knockdown cells. Data are means +/− s.e.m., from four independent experiments. **B)** *Phagosomes from p110α knockdown cells show similar levels of Rab7- RILP interaction as control cells, confirming that the Rab7 present is active.* 4 h bead phagosome lysates were immunoprecipitated with an antibody against RILP, then probed for Rab7 and RILP. Isotype IgG was added as a control for non-specific binding. Results are representative of three independent experiments. **C)** *Phagosomes isolated from p110α deficient cells are not defective in the recruitment of HOPS proteins*. Western blots of bead phagosome lysates (4 hours) were probed for the HOPS components Vps41 and Vps16. Densitometry is of means +/− s.e.m., from four independent experiments. **D)** *Flow organellometry staining of isolated phagosomes (2 h and 4 h) shows kinetics of Rab7 and Vps41 acquisition.* Phagosomes were stained for Rab7 and Vps41 and analyzed by flow cytometry. The graph shows geometric means +/− s.e.m. derived from three independent experiments. Open bars  =  Phagosomes isolated from control cells, Solid bars  =  Phagosomes from p110α knockdown cells. No significant differences in Rab7 and Vps41 recruitment were seen between phagosomes isolated from control and p110α knockdown cells.

Reduced acquisition of LAMPs and lysosomal hydrolase activity by p110α knockdown cells pointed towards a defect in phagosome-lysosome fusion. In light of this, it was important to assess whether this could be explained by a block in Rab7 activation and recruitment to the maturing phagosome membrane. To address this, lysates from 4 hour phagosomes were prepared from control and constitutive p110α knockdown cells and immunoblotting was done to assess phagosomal translocation of Rab7. Surprisingly, rather than revealing a defect, Western blotting of late stage phagosomes from p110α knockdown cells showed an approximate two-fold increase in levels of Rab7 acquisition compared to control cells (p = 0.0529, Fig. 7A). Rab7 is thought to be recruited from a cytosolic pool to membranes only when activated (in GTP-bound form) [27]. Furthermore, a recent study examining Rab membrane targeting highlighted a model in which insertion of Rab proteins into membranes is mediated by membrane-bound GEFs (guanine nucleotide exchange factors). Upon spontaneous dissociation of a Rab GDI (GDP dissociation inhibitor) from a Rab protein, Rab-GTP is generated which then inserts into the membrane [28]. In light of this model, we sought to determine the activation state of the Rab7 found on phagosomes from p110α deficient cells. This was done by examining the ability of phagosome associated Rab7 to recruit its downstream effectors, RILP, and the HOPS (homotypic vacuole fusion and protein sorting) complex to phagocytic vacuoles. RILP binds to GTP-bound Rab7 and recruits dynein-dynactin motor complexes which allow phagosomes to migrate from the cell periphery towards perinuclear lysosomes [29]–[31]. The HOPS complex is a key Rab7 effector that functions to bridge Rab7 activation and SNARE-mediated endosomal membrane fusion [32]–[36]. A recent study examining subunit arrangement within the HOPS tethering complex found that the HOPS component Vps41 interacts exclusively with Rab7 in an active state [34]. In addition, Vps16 forms a subcomplex with Vps33, the HOPS component that putatively binds SNAREs [32]–[35]. To examine phagosomal recruitment of RILP, we isolated 4 h late stage bead phagosomes from control and p110α deficient cells, and immunoprecipitated phagosome lysates with an antibody against RILP. Probing of the immunoprecipited samples with an antibody against Rab7 revealed that this protein formed complexes with RILP in phagosomes isolated from p110α knockdown cells in a manner that was indistinguishable from that of phagosomes from control cells (Fig. 7B). When phagosome lysates were immunoblotted for the HOPS components Vps16 and Vps41, when compared with control vacuoles, no significant differences in the recruitment of either Vps16 or Vps41 to phagosomes from p110α deficient cells were found (Fig. 7C). Taken together, these findings indicate that the Rab7 present on phagosomes from p110α knockdown cells was in an active state that can effectively engage downstream effectors essential for phagolysosome fusion. In addition, flow cytometric staining of phagosomes isolated from both control and p110α knockdown cells showed no significant differences in the levels of Rab7 or Vps41 (Fig. 7D), or in the cellular or phagosomal levels of the t-SNARE Vti1p and the v-SNARE VAMP7 (data not shown). These SNAREs are part of the trans-SNARE complexes which were previously shown to be involved in mediating homo- and heterotypic late endosome-lysosome fusion [37]–[45]. The presence of normal levels of key HOPS components and SNAREs indicates that the blockage in delivery of lysosomal components to phagosomes in p110α knockdown cells is not due to a lack of membrane fusion machinery, but an upstream event regulated by the PI3K p110α.

## Discussion

While there are clear lines of evidence pointing to a role for the class III PI3K, Vps34, in mediating phagosome maturation events, recent studies have suggested that other classes of these lipid kinases may contribute to this process and to endosomal trafficking in general [8], [46]–[60]. Regulation of vacuole maturation is thought to be influenced by the ligands and receptors engaged in phagocytosis, although only very recently have there been detailed studies examining the effects of specific ligand-receptor interactions on the evolving vacuole [1], [2], [46].

Previous work has indicated a need for class IA PI3Ks in phagocytosis per se of IgG-opsonized prey, and Vps34 in mediating phagosome maturation [7]. However, there are few reports to date examining the role PI3Ks play in the phagosome maturation of prey taken up by other means. A recent article directly comparing actin formation as a result of uptake by FcγR or CR3 receptors noted the formation of PI(3,4,5)P3, and the contribution of class IA PI3Ks to actin polymerization on late stage phagosomes only in the case of CR3 [46]. These results illustrate differential requirements for PI3K dependent upon the specific phagocytic receptors that are engaged. In particular, this study highlighted the role of PI(3,4,5)P3 in phagosome trafficking and the importance of class IA PI3K in development of the CR3 phagocytic vacuole. Our results indicate the involvement of a class IA PI3K in phagosome maturation, and suggest that the class IA isoform, p110α, plays a role in this process. In particular, we have shown that PI3K p110α is required for the delivery of lysosomal components to late stage phagosomes in the case of prey taken up by CR3 and non-specific phagocytic receptors. This advances the novel concept that multiple PI3K classes are involved in regulating phagosome maturation, with a requirement for class IA PI3K activity in regulating the delivery of late endosomal/lysosomal components.

At first glance, it might appear that our data were collected at relatively late time points, ranging from 2 to 6 h in some cases. In fact, this was not very unusual, as multiple studies that have used magnetic or latex beads have reported protracted phagosome maturation times [61], [62], which may be due to the indestructible nature of the prey. Nonetheless, it is important to note that we observed significant differences in the acquisition of lysosomal LAMPs, β-galactosidase, and lysosome-associated fluorescent dextran in our p110α knockdown cells between 1–2 h post phagocytosis, and that these differences were only enhanced further at later time points.

Our data indicates that phagosomes from p110α deficient cells progress normally through early maturation, as they acquire normal levels of Rab5B and EEA-1 (Fig. 3A). As it is known that EEA-1 recruitment to phagosomes is dependent on both the presence of Rab5 [60], [63]–[66] and a phagosomal pool of PI3P, it is thus not surprising that EEA-1 recruitment was observed in phagosomes from p110α knockdown cells as these cells recruited normal levels of Rab5B and showed levels of phagosomal PI3P that were similar to control cells (Fig. 2B). EEA-1 is thought to function as an early endosomal tethering molecule through its interactions with a proposed endosome fusion complex consisting of calmodulin, syntaxins 6 and 13 to bring about homotypic early endosome fusion events [64], [67]–[70]. In addition to normal Rab5B and EEA-1 recruitment, these phagosomes also showed abundant levels of Rab7 (Fig. 7A), as well as the Rab7 effectors RILP and HOPs components Vps16 and Vps41 (Fig. 7B and C). Despite this evidence for phagosomal accumulation of Rab7 competent to recruit its downstream effectors, p110α deficient cells showed clear defects in the delivery of late endosomal/lysosomal components (LAMPs, β-galactosidase, lysosome-associated fluorescent dextran). These findings indicated that Rab7 recruitment alone is insufficient to drive phagosome maturation towards phagolysosome fusion. Given that our results indicate that Rab7 by itself cannot bring about phagolysosome fusion in the absence of p110α, clearly more work needs to be done to examine the possibility that additional factors modulated by this PI3K also contribute to phagosome-lysosome interactions. Although we saw a modest and statistically significant decrease in PI(3,4,5)P3 production in our p110α deficient cells (Fig. 2B), this did not appear to be sufficient to account for the magnitude of the deficit in late endosome/lysosome interactions we observed (Figs. 3B, 4, 5). This suggests that the presence of p110α itself may be required at the phagosomal membrane in order to mediate its effects on maturation. In support of this, we found that p110α was recruited to phagosomes during the course of normal maturation, and that this was significantly abrogated in our knockdown cells (Fig. 2A).

Defective delivery of LAMP-1 and LAMP-2 to phagosomes in p110α deficient cells (Figs. 3B and 4A) indicated a block in interactions with late endosomal/lysosomal compartments. This block was not limited to membrane proteins alone, as recruitment of the luminal hydrolase, β-galactosidase, was also significantly impaired (Fig. 5). Confocal fluorescence microscopic analysis of endosomal trafficking in p110α knockdown cells showed no defects in endosomal localization or biosynthetic trafficking of LAMP-1 (data not shown) thus eliminating this as a possible explanation for defective recruitment of LAMPs to phagosomes. Furthermore, these imaging results were consistent with a previous report in which it was found that LAMP sorting from the TGN was not sensitive to wortmannin, leading to the conclusion that this process is PI3K independent [71]. While we hypothesize that the abrogation of LAMP-1 and LAMP-2 delivery to phagosomes in p110α deficient cells reflected a block in phagosome-late endosome/lysosome interactions, we cannot rule out the possibility that LAMP-1 and LAMP-2 themselves may also contribute to the maturation process *per se*. Indeed, recent studies have indicated that these late endosomal/lysosomal membrane glycoproteins may be required for the fusion of lysosomes with phagosomes [72], [73]. Thus, it may be that the defect of phagosome-lysosome fusion resulting from p110α deficiency may be exacerbated by the lack of initial delivery of LAMP proteins that promote further phagosome-lysosome interactions. Future work involving the overexpression and engineered phagosomal delivery of LAMP proteins in p110α knockdown cells should allow us to clarify the precise contributions of these membrane glycoproteins to phagosome maturation, and how this correlates with PI3K p110α activity.

Notably, defective phagosomal acquisition of β-galactosidase did not coincide with a defect in cathepsin D delivery, despite the fact that these hydrolases share the mannose-6-phosphate receptor mediated pathway for endosomal targeting [6], [74]–[77]. Taken together, these findings suggest that the block in delivery of lysosomal proteins to phagosomes is most likely explained by a selective defect in the fusion of late lysosomes with the phagosome, rather than by abnormal biosynthetic sorting of these proteins. This conclusion is supported by our confocal microscopy imaging results which showed that phagosomes in p110α knockdown cells were unable to interact with dextran-loaded lysosomes (Fig. 4B), thus indicating defective phagolysosome fusion.

Despite defects in LAMP protein and lysosomal β-galactosidase acquisition, phagosomes from p110α cells still acquired and processed the protease cathepsin D, and acidified normally (data not shown). These findings prompt us to propose that the phagosomal acquisition of cathepsin D and the vacuolar H^+^- ATPase subunits required for acidification likely take place at a late phagosome stage, but prior to bulk fusion with lysosomes. Consistent with such a model, previous studies reported that v-ATPase subunits may be delivered to phagosomes early via tubular extensions of lysosomal compartments [21], [78]–[80]. An alternative possibility to consider is that phagosomes may obtain cathepsin D and v-ATPase subunits through interactions with vesicles from the trans-Golgi network. It has been previously observed that latex bead and mycobacterial phagosomes can acquire cathepsin D through vesicles from the biosynthetic pathway [81]. Consistent with this, latex bead phagosomes also contain syntaxin 6 and cellubrevin (VAMP3), two SNAREs shown to be involved in TGN to phagosome trafficking [82], [83]. Thus it is likely that the cathepsin D we found on p110α deficient phagosomes was transported there directly from the TGN. Cathepsin D is a major component of lysosomes, and its biosynthetic transport involves cycling through acidic late endosomes [6], [77], [84] where the initial proteolytic step of removing the pro-peptide to produce the active intermediate form is believed to take place. We detected normal levels of this active intermediate form of cathepsin D in late phagosome lysates from p110α knockdown cells (Fig. 6). This suggests that despite a deficiency in p110α, phagosomes from these cells gained access to cathepsin D either through direct TGN to phagosome transport, or by interactions with late endsomes. That phagosomes from p110α deficient cells were capable of interacting readily with late endosomes was supported by the abundant presence of Rab7 on these phagosomes (Fig. 7A) which has been shown to be required for cathepsin D delivery to phagosomes [85], [86]. Moreover, the Rab7 that we detected on phagosomes from p110α deficient cells was in an active conformation since both RILP and Vps41 were recruited to these organelles (Fig. 7B and C), and both of these effectors exclusively recognize GTP-bound Rab7 [29], [31], [34]. However, despite our observation of the presence of cathepsin D in phagosomes from p110α deficient cells, we detected markedly reduced amounts of β-galactosidase or LAMPs, which also share a similar late endosome/lysosome subcellular localization (Figs. 3B, 4A, and 5). In addition, we also saw a significant decrease in phagosome interactions with lysosomes loaded with fluorescent dextran (Fig. 4B). Taken together, these results indicate that p110α is required for phagosome interactions with late endosomes/lysosomes, and that the presence of cathepsin D in these phagosomes most likely comes from direct TGN to phagosome transport.

Noteworthy, is the fact that Vps41 is a component of the HOPS complex. The multisubunit HOPs complex is made up of the class C Vps proteins Vps11, Vps16, Vps18, and Vps33 (which are also found in the endosomal CORVET complex), as well as the Rab7 effectors Vps39 and Vps41 (only found in the HOPS complex) [87], [88]. This complex serves to tether membranes together for fusion, and recent evidence points to a specific role in bridging Rab7 activation and SNARE binding [32]–[36]. Our immunoblot data of HOPS protein recruitment specifically addresses recruitment of the vacuolar HOPS complex to phagosomes as opposed to endosomal CORVET, as we probed for Vps41, which is only found in the HOPS complex. As shown in Fig. 7C, we found no significant differences in recruitment of HOPS complex to phagosomes from p110α deficient cells.

Although we did not detect any changes in HOPs complex recruitment to phagosomes in p110α knockdown cells, our data does not indicate whether this complex was in an active state competent to mediate SNARE activation. Interestingly, it has been documented in yeast that increasing the levels of Ypt7-GTP (the yeast homologue of mammalian Rab7), results in inhibition of the activity of Yck3, a vacuolar casein kinase. Yck3 positively regulates HOPS complex mediation of membrane fusion by phosphorylating the HOPs component, Vps41 [89]–[91]. Our immunoblot data of late stage phagosomes from p110α deficient cells showed nearly a two-fold increase in Rab7 levels compared to control cells (Fig. 7A). This suggests the possibility of a negative feedback loop involving inhibition of the mammalian ortholog of Yck3 by excess Rab7 and downregulation of HOPS activity. The Western and flow organellometry data showing the presence of both Vps41 and Vps16 on phagosomes from p110α deficient cells (Fig. 7C and D) suggests that there is no deficiency in HOPS complex recruitment; however, we did not examine whether this complex was able to activate SNARE assembly. The apparently normal recruitment of HOPS components, and our observations of similar levels of SNARE proteins on phagosomes isolated from control and knockdown cells, suggests that p110α deficient phagosomes should be at least partially competent to undergo membrane fusion events with late endosomes/lysosomes. On the other hand, defective delivery of LAMPs, β-galactosidase, and lysosome-associated fluorescent dextran to these phagosomes clearly indicates that there was a defect in late endosomal/lysosomal content delivery in p110α knockdown cells. Whether this was related to a lack of HOPS complex activation and SNARE priming for membrane fusion is presently unclear. Future work studying membrane fusion capability involving reconstitution of SNARE, HOPS, and phagosome components isolated from p110α cells will be required to examine how each of these specific factors contribute to the deficient phagolysosome fusion phenotype in these cells.

The findings reported above identify a specific role for the PI3K p110α in influencing phagosome - lysosome fusion. The phenotype of p110α deficient cells appears not to involve abnormal interactions with the TGN or abnormal biosynthetic sorting and most likely involves fusion of late phagosomes with late endosomes/lysosomes. The regulatory node controlled by PI3K p110α is likely located parallel to Rab7 acquisition on the phagosome membrane. This conclusion is based upon our having shown that although phagosomes isolated from p110α knockdown cells contained Rab7 capable of recruiting important effectors for membrane fusion (RILP, and HOPS component Vps41), a block in the delivery of late endosomal/lysosomal components (LAMPs, β-galactosidase, fluorescent dextran) was nevertheless observed. This interpretation is supported further by a previous finding that phagosomes in cells treated with the class I and III PI3K inhibitor wortmannin also had normal recruitment and activation of Rab7 and RILP, despite showing a profound inability to fuse with late endosomes/lysosomes [8]. The latter findings and those we are reporting here provide evidence that phagosome maturation requires an additional PI3K-mediated step in addition to Rab7 activity, and that Rab7 acquisition and activation are not sufficient to ensure maturation proceeds to completion. In addition, we found that the delivery of cathepsin D and acidification of phagosomes occurred independently of the acquisition of LAMPs and β-galactosidase. Thus, cathepsin D and vacuolar ATPase subunit recruitment may occur from direct TGN to phagosome transport. Taken together, these findings allow for development of a model of phagosome maturation in which phagosomes formed by ingestion of prey via certain phagocytic receptors recruit Rab5 and EEA-1 independently of p110α PI3K activity. Rab7 and its downstream effectors are then recruited to phagosomes. However, acquisition of functional Rab7 is by itself not sufficient for mediating fusion of the maturing phagosomes with late endosomes/lysosomes, which requires p110α activity. In conclusion, the data show that PI3K p110α plays a pivotal role in this process, and in its absence, completion of phagosome maturation does not occur.

## Materials and Methods

### Construction of p110α Knockdown Cells

The generation of constitutively silenced p110α knockdown and control THP-1 cells (ATCC TIB-202) was described previously [16]. In order to generate an inducible system for p110α knockdown using distinct shRNA sequences, a pTRIPZ construct encoding a shRNA targeting p110α was purchased from Open Biosystems (Thermo Scientific). This vector allows for the expression of shRNA after doxycycline induction. pTRIPZ lentivirus was produced and THP-1 cells were transduced as described previously [16].

### Cell Culture and Induction of p110α Knockdown

All cells were maintained in RPMI media (Stem Cell Technologies) supplemented with 10% fetal bovine serum (Gibco), and 2 mM L-glutamine (Stem Cell Technologies). Twenty-four hours prior to phagosome maturation assays, constitutively silenced p110α knockdown and control cells were given 10 ng/mL of phorbol 12-myristate 13-acetate (PMA, Sigma-Aldrich) and seeded onto 6-well culture plates (2×10^6^/well) for differentiation at 37°C/5% CO_2_. Three hours prior to feeding prey, cells were washed once with warm media and allowed to rest. For pTRIPZ transduced THP-1, cells were seeded into 6-well culture plates with 10 ng/mL of PMA and 2 µg/mL of doxycycline. Cells were allowed to differentiate for 24 h, then washed once with warm media, and subsequently given fresh media with 2 µg/mL of doxycycline for an additional 24 h prior to experimentation.

### Phagocytosis Assays

#### Alexafluor 633 labelling of *M*. *smegmatis* and infection

Mycobacterium smegmatis mc^2^155 was cultured from frozen glycerol stocks in LB media. Cultures were shaken overnight at 37°C to log phase, then the optical density at 580 nm was measured to determine cell titer. The required number of bacteria for an MOI of 20∶1 was taken from this culture, and washed three times with Middlebrook 7H9 media (BD Biosciences) supplemented with 0.05% Tween-80, then resuspended in 200 µL of Middlebrook 7H9 with Tween-80. A final concentration of 30 µg/mL of Alexafluor 633 succinimidyl ester (-SE, Molecular Probes) was added, and the mixture was incubated at 37°C for 1 hour with rotation. Bacteria were centrifuged at 13000×g for 1 minute.

For phagocytosis assays, bacteria were then washed three times with RPMI, resuspended to give a final cell titer of 1.6E6/µL and used to infect PMA-differentiated THP-1 in culture plates. Phagocytosis was synchronized by placing cells at 4°C for 20 minutes prior to incubation at 37°C /5% CO2. Timepoints were taken by washing cells three times with PBS (without Mg2^+^/Ca2^+^, Stem Cell Technologies), resuspending cells in 1 mL of PBS with 0.2% trypan blue (used to quench fluorescence of extracellular bacteria), then pelleting the cells by centrifugation at 1500×g for 2 minutes. Cell pellets were washed three times with PBS, then resuspended and fixed in 2% paraformaldehyde (Electron Microscopy Sciences) in PBS and stored at 4°C. For quantification of bacteria uptake, samples were washed once, resuspended in PBS, and run on a flow cytometer (FacsCalibur, Becton Dickinson). Data were analyzed using FlowJo software (v8.7, Tree Star, Inc.).

### BSA-coupling and Alexafluor 633 Labelling of Magnetic Beads and Bead-treatment of THP-1 Cells

Carboxylic acid functionalized 3 µm diameter magnetic beads (COMPEL™, Bangs Laboratories) were coupled with bovine serum albumin (Fisher Scientific) as per manufacturer’s protocol (Bangs Laboratories). Washed coupled beads were then labelled with Alexafluor 633-SE as stated above for *M. smegmatis* labelling, but instead of centrifugation, beads were pulled away from supernatants with the use of a magnetic tube holder (Bangs Laboratories). PMA-differentiated THP-1cells were treated with RPMI-washed and resuspended beads at an MOI of 5∶1, phagocytosis was synchronized and timepoints were taken as described. Quantification of bead uptake was done with a flow cytometer (FacsCalibur, Becton Dickinson).

### Phagosome Marker Staining and Analysis

#### Phagosome isolation

Phagosomes containing BSA-coupled 3 µm magnetic beads were isolated from bead-fed THP-1 cells chased at the timepoints indicated. Culture plate wells containing cells were washed three times with PBS (without Mg2^+^/Ca2^+^), then cells were resuspended in PBS and pelleted at 330×g at 4°C for 10 minutes. Cell pellets were resuspended in ice-cold homogenization buffer (0.25 M sucrose, 10 mM HEPES, pH 7.4, protease inhibitor cocktail (Roche) in PBS), and then lysed by passage through a 27 1/2 gauge needle 80 times. Cell lysis was confirmed by placing a sample of the homogenate under a light microscope and determined to be >99%. For isolation of bead phagosomes, homogenates were transferred to 12×75 mm round bottom culture tubes (VWR) and placed on ice into a cold magnetic cell separating block (EasySep®, Stem Cell Technologies) for 3 minutes. Isolated bead phagosomes were washed three times with ice-cold PBS, then fixed with 2% paraformaldehyde in PBS. Phagosomes were stored at 4°C until staining.

#### Markers staining

Antibodies against LAMP-1 and LAMP-2 were obtained through the Developmental Studies Hybridoma Bank. Anti-Rab5B, EEA-1, Vti1p, and VAMP7 and Actin were purchased from Santa Cruz Biotechnology. Antibodies against PI3P and PI(3,4,5)P3 were purchased from Echelon Biosciences Inc. Antibodies against Vps16 and Vps41 were purchased from Novus Biologicals. Cy5-conjugated secondary antibodies were from Applied Biological Materials, and mouse, goat, and rabbit IgG isotype controls were purchased from Sigma-Aldrich. Briefly, phagosomes were permeabilized and blocked with blocking buffer (1% BSA in PBS containing 0.05% Tween-20 and 0.2% saponin) for 30 minutes at room temperature, followed by pelleting at 600×g for 5 minutes. Then phagosomes were incubated with primary antibodies and isotype controls in blocking buffer for 1–3 hours at room temperature. Phagosomes were then washed three times with PBS containing 0.05% Tween-20 (PBS-T), and incubated with secondary antibody in blocking buffer for 1 hour at room temperature. After washing three times with PBS-T, phagosomes were resuspended in PBS, then analysed on a FacsCalibur flow cytometer. The resulting data was analyzed with FlowJo software (v8.7, Tree Star, Inc).

### Confocal Microscopy

Control and p110α knockdown cells were seeded onto coverslips and differentiated overnight with 10 ng/mL PMA. Cells were then washed once with complete medium and allowed to rest for 4 hours prior to bead treatment as described above. Cells were allowed to ingest beads for 15 min after which they were washed three times with warm PBS and replenished with complete medium. After 2 h of incubation, cells were fixed with 2% paraformaldehyde, washed three times with PBS, then permeabilized with 0.1% Triton X-100 in PBS at room temperature for 10 min. Following washes with PBS, permeabilized cells were incubated with LAMP-1 antibodies (Developmental Studies Hybridoma Bank) overnight at 4°C, followed by Alexafluor 555 - conjugated anti-mouse secondary (Molecular Probes) for 1 h at room temperature. After washing with PBS, coverslips were mounted onto Prolong Gold Antifade reagent with added DAPI stain (Molecular Probes), and Z-stacks of cells were imaged using a Leica DMIRE2 inverted microscope equipped with a SP2 AOBS laser scanning head. Images were processed using the MacBiophotonics version of the Image J software (National Institutes of Health). For Texas Red dextran experiments, cells were seeded onto coverslips as described, with the addition of 0.25 mg/mL of Texas Red dextran (Molecular Probes) to the culture medium. Cells were then incubated overnight, washed three times with warm PBS and given fresh medium. This was followed by a 4 h incubation in order to chase fluorescent dextran into the lysosomal compartment. Cells were then fed beads, allowed to phagocytose for 15 min, washed, given fresh medium, and incubated for 2 h prior to fixation. At least 100 phagosomes were counted from three independent experiments for each set of experiments.

### β-galactosidase Assays

Assays were done as per Yates *et al*. [80]. The red fluorescence β-galactosidase substrate, C_12_RG, was used for experiments done with constitutive p110α knockdown and control cells, while the green substrate, C_12_FDG was used for pTRIPZ transduced cells (Molecular Probes). *M. smegmatis* and 3 µm BSA-coupled magnetic beads that had been previously labelled with Alexafluor 633-SE were subsequently incubated with C_12_RG or C_12_FDG for 1 h at 37°C, then washed three times with RPMI. Prey was then resuspended in RPMI and used to infect PMA-differentiated THP-1 at their respective MOIs (20∶1 for *M. smegmatis*, and 5∶1 for beads).

### Bead-phagosome Lysate Preparation, Immunoprecipitation and Western Blotting

Four hour phagosomes containing magnetic beads were isolated from PMA-differentiated THP-1 cells as described above, with the exception that instead of fixation, PBS-T was used to resuspend phagosomes, followed by centrifugation at 1900×g for 5 minutes. Pellets were resuspended in 1x SDS loading buffer (63 mM Tris-Cl, pH 6.8, 715 mM 2-mercaptoethanol, 2% SDS, 20% glycerol) and boiled prior to running in 10% or 12.5% SDS-PAGE. Following transfer onto nitrocellulose, blots were probed with antibodies against Rab7 (Abcam), Cathepsin D (G-19 clone, Santa Cruz Biotechnology), Vps16 and Vps41 (Novus Biologicals), and p110α (Cell Signaling Technology). For immunoprecipitation experiments, phagosomes were isolated as described, but were then lysed in lysis buffer (20 mM Tris pH 8.0, 137 mM NaCl, 1% glycerol, 1% Triton X-100, 1 mM EDTA, 1 mM PMSF, and protease inhibitor cocktail mix (Roche) as described [92]. Phagosomes were kept on ice for 10 min, followed by centrifugation at 13,000×g for 10 min at 4°C. All subsequent steps were conducted either on ice or at 4°C. Lysate was precleared by incubation with Protein A agarose (Santa Cruz Biotechnology) for 10 min, then centrifuged at 13,000×g for 1 min to remove the Protein A agarose. Cleared lysate was incubated with 2 µg/mL of antibody against RILP (Santa Cruz Biotechnology) overnight, followed by incubation with Protein A agarose for an additional 3 h. Lysate was centrifuged at 13,000×g for 1 min, and washed five times with lysis buffer. Samples were then boiled in 2× SDS loading buffer and loaded onto 10% SDS-PAGE for immunoblotting with an antibody against Rab7. All blots were developed using Supersignal West Pico and Femto Chemiluminescent Substrate (Thermo Fisher Scientific).

### Statistical Analyses

All statistical analyses were done with GraphPad Prism software. Paired t-tests were done on all data to compare p110α knockdown with control cells at each timepoint. Results were considered significant at p<0.05.
